# CancerWatch: accelerating Europe’s cancer intelligence

**DOI:** 10.2340/1651-226X.2026.46506

**Published:** 2026-09-17

**Authors:** Claudine Backes, Max Xhauflair, Gijs Geleijnse, Maria Dolores Chirlaque, Manola Bettio, Mario Šekerija, Liesbet van Eycken, Matthijs Sloep, Esteve Rodon Navarro, Dafina Petrova, Yasmin Cura, Maria-José Sánchez, Giske Ursin

**Affiliations:** a Registre National du Cancer du Luxembourg (RNC), Strassen, Luxembourg; b Cancer Epidemiology and Prevention Group (EPICAN), Department of Precision Health (DoPH), Luxembourg Institute of Health (LIH), Strassen, Luxembourg; c Department of Research & Development, Netherlands Comprehensive Cancer Organisation (IKNL), Utrecht, The Netherlands; d Centro de Investigación Biomédica en Red de Epidemiología y Salud Pública (CIBERESP), Madrid, Spain; e Department of Epidemiology, Regional Health Council, IMIB-Arrixaca, Murcia University, Murcia, Spain; f European Commission, Joint Research Centre (JRC), Ispra, Italy; g Croatian Institute of Public Health, Zagreb, Croatia; h Andrija Stampar School of Public Health, University of Zagreb School of Medicine, Zagreb, Croatia; i Belgian Cancer Registry, Brussels, Belgium; j Catalan Institute of Oncology, Catalonia, Spain; k Escuela Andaluza de Salud Pública, Granada, Spain; l Instituto de Investigación Biosanitaria ibs.GRANADA, Granada, Spain; m University Hospital Virgen de las Nieves, Granada, Spain; n The Cancer Registry of Norway, Norwegian Institute of Public Health, Oslo, Norway; o Department of Nutrition, Institute of Basic Medical Sciences, University of Oslo, Oslo, Norway; p Department of Preventive Medicine, University of Southern California, Los Angeles, CA, USA

**Keywords:** Neoplasms (D009369), epidemiology (D004813), registries (D012042), population surveillance (D011159), public health surveillance (D062486), data accuracy (D000068598), health information interoperability (D000073892)

## CancerWatch: strengthening Europe’s cancer intelligence

Cancer remains one of Europe’s most pressing public health challenges, with 2.7 million new cases and 1.3 million deaths estimated each year [1]. By 2050, cancer incidence and mortality are projected to rise by 20.4% and 34.6% respectively, while per-capita cancer spending across the EU27 is forecast to increase by 59% in real terms over the same period [2, 3]. Population ageing, persistent inequalities and rising costs are expected to increase pressure on European health systems [4, 5]. Assessing this growing burden and evaluating the effectiveness of cancer control therefore require timely, comparable and robust population-based evidence.

In 2021, the European Commission launched Europe’s Beating Cancer Plan (EBCP), supported by €4 billion to strengthen cancer control [6]. Its four action areas cover prevention, early detection, diagnosis and treatment, and quality of life of all patients and survivors. However, a recent assessment by the European Court of Auditors found that the EBCP monitoring framework lacks quantified targets, milestones and indicators, highlighting the need for harmonised and comparable population-based data to assess progress and impact [7].

## Population-based cancer registries: a foundation for cancer intelligence

Population-based cancer registries (PBCRs) provide systematic, continuous and standardised real-world evidence on cancer across Europe. Their cancer collection draws on multiple data sources, harmonised coding and standardised analytical methods that follow international recommendations, supporting quality and comparability across populations [8–11].

Unlike hospital-based or medico-administrative datasets, PBCRs capture core clinical information on all incident cancers within a defined population, irrespective of where patients are diagnosed or treated. This population-wide coverage is essential for more comprehensive and less selection-prone assessment of patient-centred cancer incidence, survival and other population-level outcomes. The Lancet Commission identified PBCRs as essential for evaluating cancer control policies and supporting population-based research [12]. The reliability of PBCR data is commonly assessed through four competing dimensions: completeness, validity, comparability and timeliness [13, 14]. Maintaining high performance across all four remains challenging, particularly as demand for more timely cancer data increases.

Beyond surveillance, PBCRs support population-based research on cancer aetiology, survival, inequalities and health-system performance. PBCRs are also essential for monitoring key cancer-control objectives across the entire cancer patient pathway. For example, they enable evaluation of organised screening programmes through indicators such as stage at diagnosis, interval cancers, survival and mortality, providing evidence of population-level effectiveness. Similarly, the target that 90% of eligible patients have access to a Comprehensive Cancer Centre by 2030 requires robust population-based data to assess the quality of care and outcomes across the entire cancer population, including patients diagnosed or treated outside accredited centres. The Europe-wide certification framework for these centres, currently being implemented, reinforces the need for harmonised population-based monitoring of cancer care, outcomes and inequalities [15].

However, the European landscape is heterogeneous. Unlike mortality statistics, which are supported by an EU-level regulatory framework, cancer registration remains a national responsibility with no equivalent regulatory basis. European countries differ in their capacity to generate timely and comparable population-based cancer intelligence because of differences in governance, healthcare systems, legal frameworks, workforce capacity, digital infrastructure and data availability. The depth of routinely collected clinical and treatment data varies considerably across registries, with high-resolution information often requiring additional data linkage or specialised studies. Addressing these disparities requires common methodologies adapted to local contexts, supported by coordinated implementation.

PBCR outputs depend on a specialised workforce, yet recruitment and retention remain barriers to complete and timely data collection [16]. Well-governed digitalisation and artificial intelligence (AI) across the cancer data lifecycle offer opportunities to improve efficiency and reduce workload. Automation remains uneven: 52% of European registries manually verify all automated outputs, and 74% report that algorithms for complex coding tasks require further development [17]. Reliable real-world comparisons across Europe require harmonisation of data definitions, coding and analytical methods [18–21]. Digital transformation offers opportunities to improve completeness, validity, timeliness and the depth of available clinical information, but cannot by itself ensure comparability; common standards, clear governance and robust quality assurance mechanisms remain essential [22–26].

## PBCRs in Europe

Most European countries operate PBCRs with regional or national coverage, governed by public health authorities, hospitals or other governmental organisations. Some have long-established registries with integrated digital workflows, while others still rely on labour-intensive manual processes because of technical, organisational or legal barriers to data access and sharing [24]. Established in 1990, the European Network of Cancer Registries (ENCR) supports standardised data collection, comparability and collaboration among PBCRs across Europe. Its Secretariat is operated by the European Commission’s Joint Research Centre (JRC), which, in collaboration with the Directorate-General for Health and Food Safety, developed the European Cancer Information System (ECIS), a web-based platform that disseminates harmonised cancer indicators based on quality-assured PBCR data [16, 17, 27].

The ENCR member list covers over 100 PBCRs, representing approximately 75% of the EU population [28]. While the ECIS routinely reports incidence and mortality, survival and prevalence estimates are derived from EUROCARE, based on cancer diagnoses from 2000–2007 and up to 2014, respectively [1, 29]. Stage-specific indicators, essential for evaluating early detection, treatment and inequalities in cancer outcomes, are not yet routinely published at European level because standardised stage data remain difficult to collect [30]. Work is underway within the JRC–ENCR collaboration to improve the availability and consistency of stage data across European PBCRs, providing an important foundation for future population-based stage indicators [27, 31].

A key challenge for PBCRs is reconciling the demand for more timely data with the methodological rigour needed for high-quality registration within existing resources. For example, the forthcoming introduction of the International Classification of Diseases for Oncology, 4th edition, highlights the need to modernise coding systems and supporting technologies [9]. Implementation will therefore require common coding guidance, training, validation and quality control in addition to technical upgrades. Together, these challenges point to the need for a coordinated approach that strengthens registry capacity while modernising the tools and processes used to generate cancer intelligence.

## The CancerWatch Joint Action: strengthening European cancer intelligence

**Figure 1:**
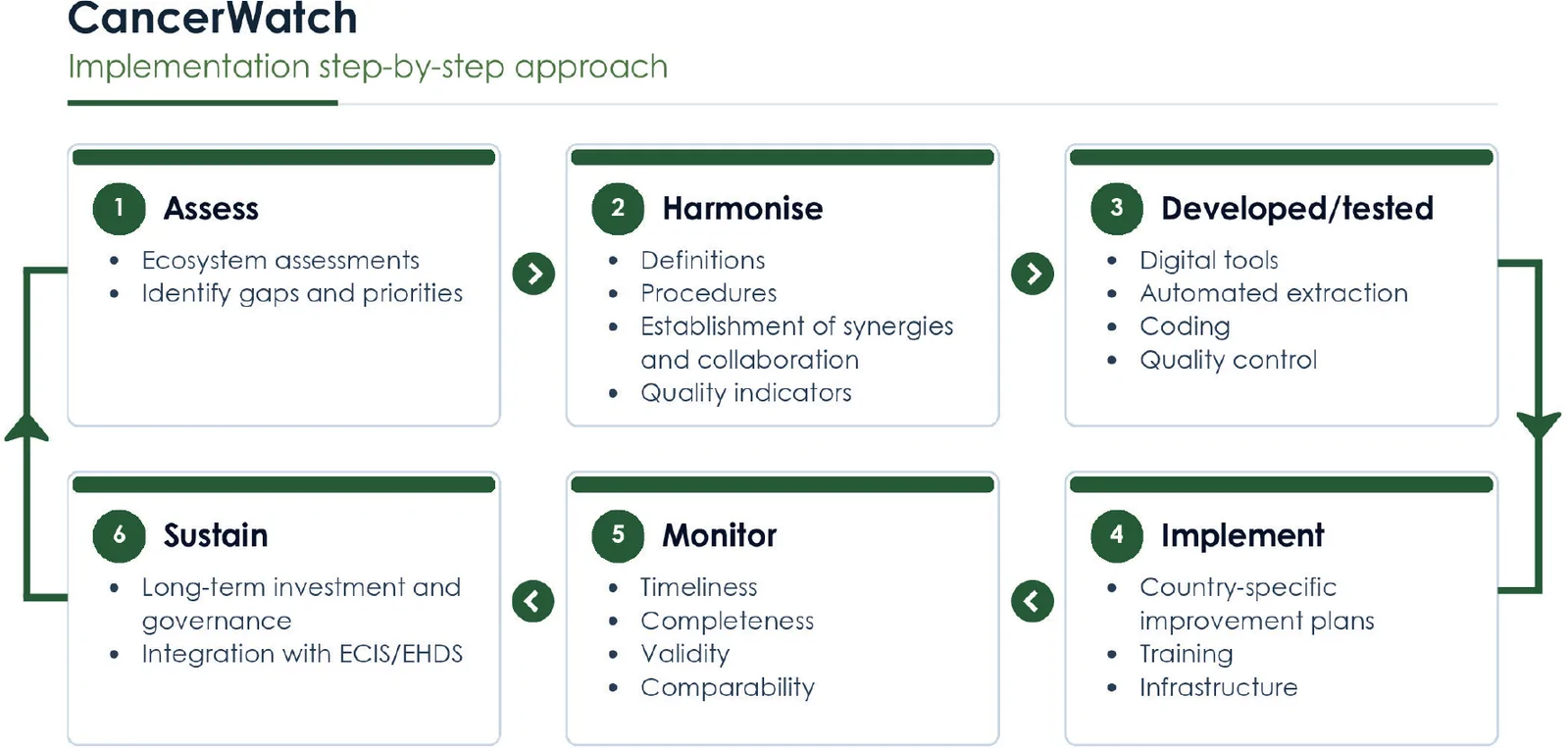
The Cancer Watch implementation framework.

CancerWatch provides a coordinated European implementation framework to strengthen cancer intelligence. CancerWatch follows a step-by-step approach, beginning with an assessment of gaps and priorities, followed by harmonisation of definitions and procedures, development and testing of digital tools, country-specific implementation and training, and monitoring of progress and impact (Figure 1). Long-term sustainability depends on sustained national investment, clear governance arrangements and continued collaboration among national and European stakeholders.

The Joint Action runs from September 2025 to August 2028 and aims to improve the quality and timeliness of population-based cancer data contributing to the ECIS.

With 92 partners from 29 countries, CancerWatch strengthens the interface between national PBCRs and the ECIS. Its activities are organised across seven work packages covering coordination, registry operations, digitalisation, data quality and availability, sustainability, and the development of cancer data insights and indicators.

## Innovation across the cancer data lifecycle

The 3-year CancerWatch Joint Action addresses the cancer data lifecycle from case identification and data capture through validation and indicator production. During the first year, CancerWatch conducted a large-scale survey of 192 European cancer registries, achieving an 91,1% response rate [32]. The survey assesses organisational capacity, capabilities, data flows and practices, including governance, population coverage, data sources, automation, quality control, data security, workforce capacity and analytical expertise. It identifies common challenges and country-specific gaps and informs the development of a common framework and practical tools to improve data quality, timeliness, interoperability and comparability, while taking national legal and organisational contexts into account.

CancerWatch is also developing and testing digital solutions across the PBCR workflow, including automated extraction from health information systems, AI-assisted processing of pathology reports, interoperability with electronic health records, and automated support for case identification and coding [33–35]. These tools aim to reduce workload, improve completeness and accelerate reporting while maintaining validity through appropriate quality assurance and expert oversight. A key objective is to reduce the time required for PBCRs to report incidence data to the ECIS to less than 2 years. Implementation will depend on technical, legal and organisational interoperability among registries, hospitals and other data holders.

## Cancer intelligence in the European Health Data Space

The European Health Data Space (EHDS) will provide an important opportunity to strengthen the use of cancer data for research, innovation and policy. CancerWatch supports the preparation of PBCRs for integration within this evolving framework by strengthening documentation, quality assurance procedures, interoperability and adherence to FAIR (Findable, Accessible, Interoperable, Reusable) data principles [36]. EHDS integration requires not only data availability but also transparent metadata, documented data quality, interoperability and clear governance [37]. CancerWatch’s quality-control framework is designed to support alignment with emerging EHDS requirements and incorporates harmonised documentation and approaches developed through the EU-funded Horizon project QUANTUM (‘Quality, Utility and Maturity Measured’), which developed a European quality label for health datasets. CancerWatch incorporates QUANTUM-based quality labelling, automated plausibility checks, incidence–mortality comparisons and adherence to FAIR data principles [38, 39]. The CancerWatch Quality Checking Software is being expanded to support the publication of meaningful metadata in the EHDS catalogue, addressing both EHDS requirements and the specific needs of cancer registry data.

Beyond timeliness and quality, by the end of 2026, CancerWatch will provide updated survival and prevalence indicators in the ECIS and support their timely dissemination. Standardised tools are enabling PBCRs to compute these indicators locally while sharing harmonised outputs with the ECIS.

## Sustainability beyond CancerWatch

For CancerWatch innovations to have lasting impact, PBCRs must be embedded in sustainable national and international structures. As health systems remain a national competence, the success of EU-funded initiatives also depends on national follow-up, governance and funding [7, 31, 40].

National cancer registration mandates should support complete population coverage and timely data submission to the ECIS in line with ENCR recommendations. A June 2026 G7 call to action on cancer also urged stronger links between national registries and cross-border data collaboration while respecting national competences [41]. Stable national funding, complemented by European coordination including the JRC and the ECIS, will be essential to sustain timely and high-quality cancer information. Legal and governance frameworks should reinforce the coordinating role of PBCRs.

The eCancer Working Group, established under the Cancer Subgroup of the European Commission’s Public Health Expert Group, recommends establishing National Cancer Data Nodes within the UNCAN.eu network, with cancer registries identified as leading candidates to host these nodes [42]. These nodes would provide a national point of coordination, linking cancer data from clinical, registry and research sources. Given their population-based design, expertise in data quality assurance, and established use of international coding and classification standards, PBCRs are well placed to contribute to this framework and support comparable cancer intelligence across countries within the EHDS.

Strengthening PBCRs at national and EU levels will be important for monitoring cancer burden, evaluating policy impact and supporting evidence-based decision-making under the EBCP [16, 43]. In countries where registries are underdeveloped or absent, investment in governance, workforce and digital infrastructure is essential to improve data completeness and timeliness [4, 27, 44]. Such investment should also support the progressive development of clinical data depth, including the capacity to link registry data with relevant clinical, treatment, pathology and other complementary data sources where appropriate.

CancerWatch builds on the long-standing collaboration between PBCRs, the ENCR and the JRC, whose complementary roles in cancer surveillance, methodological harmonisation, quality assurance, data integration and indicator production form the backbone of Europe’s cancer intelligence system. By bringing together countries, PBCRs, the ENCR, the JRC and the European Commission around shared priorities, standards, tools and measurable objectives, CancerWatch provides a common European implementation framework while respecting national responsibility for cancer registration.

Ultimately, the success of CancerWatch will be measured not only by the tools or outputs delivered during the Joint Action, but also by their sustained implementation within national cancer registration systems. Its lasting impact will depend on political commitment, national investment, continued European coordination and integration of its approaches into routine practice. In this way, CancerWatch can help ensure that high-quality population-based cancer data are translated into timely, comparable and actionable cancer intelligence – and ultimately into better decisions and stronger cancer control across Europe.

## Data Availability

No datasets were generated or analysed for this manuscript.
